# Genome Sequence of Thermophilic *Bacillus licheniformis* Strain 3F-3, an Efficient Pentose-Utilizing Producer of 2,3-Butanediol

**DOI:** 10.1128/genomeA.00615-14

**Published:** 2014-06-26

**Authors:** Lixiang Li, Yu Wang, Kai Wang, Kun Li, Cuiqing Ma, Ping Xu

**Affiliations:** a State Key Laboratory of Microbial Metabolism, Shanghai Jiao Tong University, Shanghai, China; b State Key Laboratory of Microbial Technology, Shandong University, Jinan, China

## Abstract

*Bacillus licheniformis* strain 3F-3 is an efficient pentose-utilizing producer of platform chemical, 2,3-butanediol. Here we present a 4.1-Mb assembly of its genome. The key genes for pentose utilization, regulation, and metabolism of 2,3-butanediol were annotated, which may provide further insights into the molecular mechanism of 2,3-butanediol production from biomass pentose.

## GENOME ANNOUNCEMENT

2,3-Butanediol (2,3-BD) is an important platform biochemical, which can be used to produce valuable derivatives such as methyl ethyl ketone and 1,3-butadiene (1, 2). It can also be used to produce renewable polyesters (3). Recently, microbial 2,3-BD production has attracted great attention worldwide, as processes used for renewable biomass are promising routes for developing a low-carbon economy and a gateway to a more sustainable future (1, 4). Biomass lignocellulose-derived sugars, including glucose, xylose, and arabinose, are considered economically attractive carbohydrates for the large-scale fermentation of important platform chemicals. However, few organisms effectively convert pentose to 2,3-BD at high levels (2). In addition, most pentose-utilizing organisms lack thermal tolerance, which limits their fermentation activity at temperatures above 43°C (5). Thermophilic fermentation reduces the risk of bacterial contamination, making it more efficient and cost-effective (6).

Recently, *B. licheniformis* 10 to 1-A has been reported to produce 2,3-BD from glucose with high concentrations and productivity at 50°C (7, 8). It also produces 2,3-BD from xylose, but the productivity is low. However, the *B. licheniformis* strain 3F-3 (CCTCC M 2012253) isolated from soil samples is an efficient pentose-utilizing 2,3-BD producer. Our unpublished results showed that strain 3F-3 produces 2,3-BD from xylose with high productivity (>1.0 g liter^–1^ h^–1^) and high concentrations (>60 g liter^–1^) at a temperature of 50°C. It also produces 2,3-BD from glucose, arabinose, corncob molasses, and corn straw hydrolyte with high concentrations of diols (2,3-BD and acetoin) (>50 g liter^–1^).

Here, we present the draft genome sequence of strain 3F-3, which was obtained using the Illumina HiSeq 2000 system. All the reads for 3F-3 were assembled into 40 contigs using VELVET (9). The genome annotations were performed by the RAST server (10). The functional descriptions were determined using Clusters of Orthologous Genes (11). The genome sequence of strain 3F-3 consists of 4,194,413 bases with a G+C content of 46.2%. According to the annotation of the RAST system, there are 4,556 protein-coding sequences (CDSs) in the genome, among which 2,140 CDSs (47%) were assigned putative biological functions. A total of 481 subsystems were determined using the RAST server in the genome, and this information was used to by the RAST system to construct the metabolic network. Based on carbohydrate metabolism analysis, 12 CDSs for the metabolism of pentose sugars were annotated, which are related to the pentose metabolite pathway, including xylose/arabinose isomerase, ribulokinase, and ribulose-5-phosphate-4-epimerase. The transketolase/transaldolase pathway, instead of phosphoketolase, was predicted in the genome, implying that strain 3F-3 utilizes pentose more efficiently. There are key enzymes (cellulase, alpha-xylosidase, beta-glucosidase, 1,4-beta-cellobiosidase, and alpha-amylase) for utilization of biomass in strain 3F-3, indicating that strain 3F-3 may be a good candidate for 2,3-BD production from biomass. The sequence contains two complete operons and key coding genes for 2,3-BD metabolism, which might provide further insights into production of 2,3-BD.

### Nucleotide sequence accession number.

The whole-genome shotgun projects have been deposited at DDBJ/EMBL/GenBank under the accession number JFYM00000000 for strain 3F-3. The version described in this paper is the first version.
